# Normative references of heart rate variability and salivary alpha-amylase in a healthy young male population

**DOI:** 10.1186/1880-6805-31-9

**Published:** 2012-04-20

**Authors:** Hiromitsu Kobayashi, Bum-Jin Park, Yoshifumi Miyazaki

**Affiliations:** 1 Department of Nursing, Ishikawa Prefectural Nursing University, Ishikawa, Japan; 2 Department of Environment & Forest Resources, Chungnam National University, Deajeon, Korea; 3 Center for Environment, Health and Field Sciences, Chiba University, Chiba, Japan

**Keywords:** heart rate variability, kurtosis, reproducibility, salivary alpha-amylase, skewness

## Abstract

**Background:**

This study aimed to present normative reference values of heart rate variability and salivary alpha-amylase in a healthy young male population with a particular focus on their distribution and reproducibility.

**Methods:**

The short-term heart rate variability of 417 young healthy Japanese men was studied. Furthermore, salivary alpha-amylase was measured in 430 men. The average age of the subjects were 21.9 years with standard deviation of 1.6 years. Interindividual variations in heart rate variability indices and salivary alpha-amylase levels were plotted as histograms. Data are presented as the mean, median, standard deviation, coefficient of variation, skewness, kurtosis, and fifth and 95th percentiles of each physiological index.

**Results:**

Mean recorded values were heart period 945.85 ms, log-transformed high frequency component 9.84 ln-ms^2^, log-transformed low frequency component 10.42 ln-ms^2^, log-transformed low frequency to high frequency ratio 0.58 ln-ratio, standard deviation of beat-to-beat interval 27.17 ms and root mean square of successive difference 37.49 ms. The mean value of raw salivary alpha-amylase was 17.48 U/mL, square root salivary alpha-amylase 3.96 sqrt[U/mL] and log-transformed salivary alpha-amylase 2.65 ln[U/mL]. Log-transformed heart rate variability indices exhibited almost symmetrical distributions; however, time-domain indices of heart rate variability (standard deviation of beat-to-beat interval and root mean square of successive difference) exhibited right-skewed (positive skewness) distributions. A considerable right-skewed distribution was observed for raw salivary alpha-amylase. Logarithmic transformation improved the distribution of salivary alpha-amylase, although square root transformation was insufficient. The day-to-day reproducibility of these indices was assessed using intraclass correlation coefficients. Intraclass correlation coefficients of most heart rate variability and salivary indices were approximately 0.5 to 0.6. Intraclass correlation coefficients of raw salivary markers were approximately 0.6, which was similar to those of heart rate variability; however, log transformation of the salivary markers did not considerably improve their reproducibility. Correlations between sympathetic indicators of heart rate variability and salivary alpha-amylase were not observed.

**Conclusion:**

Because the sample population examined in this study involved limited age and gender variations, the present results were independent of these factors and were indicative of pure interindividual variation.

## Background

Heart rate variability (HRV) has been extensively studied as an index of human autonomic nervous function. Low frequency (LF) and high frequency (HF) components of HRV are considered markers of sympathetic and parasympathetic nervous activities, respectively [1]. A relationship between HRV and autonomic functions has been established by several studies [2-4].

HRV measurements can be divided into two methods: long-term and short-term measurements. In long-term HRV measurements, heart rate is typically continuously recorded for 12 to 24 hours. Because it is a non-restrained recording, subjects can continue their routine life during the measurement. Therefore, larger populations are relatively easy to evaluate in long-term HRV measurements, and some studies performed measurements in sample sizes of 1,000 individuals [5-7]. In contrast, 2- to 5-minute recordings are commonly used to measure short-term HRV [1]. In ambulatory measurements, a participant's body movements, utterances or other behaviors affect HRV. In particular, changes in posture and breathing can distinctly influence HRV [8,9], decreasing the reliability of long-term HRV measurements [10]. In short-term measurements, these artifacts are controllable. However, the sample size tends to be smaller in short-term HRV measurements because these measurements are typically performed in laboratory settings.

In recent years, salivary alpha-amylase (sAA) has attracted attention as a biomarker of sympathetic nervous activity [11]. Amylase is an enzyme related to carbohydrate digestion, and its secretion is controlled by the sympathetic-adrenal medullary system. Similar to HRV measurements, sAA can be measured non-invasively. Recent developments in the devices for monitoring sAA have extended the application of this measurement into various fields of human science.

The results of HRV and sAA measurements indicate significant individual variations. Because of these variations, these physiological measurements often produce unclear results. Therefore, understanding individual variations in these physiological indices is necessary for appropriate interpretation of human autonomic nervous function. This study aimed to elucidate normative reference values of HRV and sAA in a healthy young male population with a special focus on their distribution and day-to-day reproducibility.

## Methods

### Participants

The study consisted of 456 Japanese male students aged 20 to 29 years old (mean, 21.9 ± 1.6 years). Height was in the range of 158 to 182 cm (mean 172.1 ± 5.6 cm) and weight was between 53 and 82 kg (mean 64.5 ± 9.3 kg). All participants were non-smokers, and alcohol intake on the day before the measurement was forbidden.

The study was conducted under the regulations of the Institutional Ethical Committee of the Forestry and Forest Products Research Institute and Center for Environment, Health and Field Sciences, Chiba University, Japan. The aim and procedure of the experiment were explained to the participants, and written informed consent was obtained.

Measurements were performed in the morning before breakfast (6:30 to 7:30 a.m.). Each participant rested for 1 minute in a sitting position, and beat-to-beat intervals during spontaneous breathing were recorded for the next 2 minutes. The measurement was repeated using the same procedure one day after the first measurement to confirm intra-individual reproducibility. We measured HRV and sAA in 456 individuals; however, because of failed measurements, only 417 and 430 subjects were included in HRV and sAA analyses, respectively.

### Heart rate variability measurements

Beat-to-beat heart rate was recorded with 1-ms resolution using portable heart rate monitors (AC301; GMS, Japan). Beat detection errors or any ectopic beats were corrected or excluded from the analysis. The heart period (HP), standard deviation of normal-to-normal intervals (SDNNs) and root mean square of successive differences (rMSSDs) were calculated from the 2-minute recordings.

Spectral analysis was performed by the maximum entropy method (MEM), and autoregressive coefficients were estimated using the Burg algorithm. A fixed autoregressive model order (12th) was used for the spectral analyses according to previous studies [12,13]. The power spectrum was calculated from 0.01 to 0.40 Hz with 0.01-Hz frequency resolution. HF and LF components were calculated through integration of the power spectra of 0.15 to 0.40 Hz and 0.04 to 0.15 Hz, respectively. HF, LF and the LF to HF ratio were then converted into natural logarithms (lnHF, lnLF, and ln[LF/HF]).

### Salivary alpha-amylase measurement

sAA was measured using a hand-held amylase monitor (α-AMY; YAMAHA, Japan). This device quantifies sAA activity based on a dry chemical system. The effects of environmental temperature and pH on individual samples were automatically adjusted to reflect the effects of 37°C and pH 6.5. The accuracy of the amylase monitor has been validated previously [14]. Saliva samples from each individual were collected using a test strap. The time required for saliva collection using the strap was 2 minutes. The collected samples were immediately analyzed, and analysis was completed within a minute for each sample.

### Statistical analysis

The average of the results from the two experimental days was used for analysis of distribution characteristics. The mean, median, SD, coefficient of variation (CV; SD/mean), skewness (a measure of symmetry), kurtosis (a measure of peakedness) and fifth and 95th percentiles of the physiological indices were calculated.

Intraclass correlation coefficients (ICCs) were calculated from the results of two repeated measurements as an index of intra-individual reproducibility. Strictly speaking, ICC used in this study was ICC (1, 1) according to the classification by Shrout and Fleiss [15].

## Results

**Figure 1:**
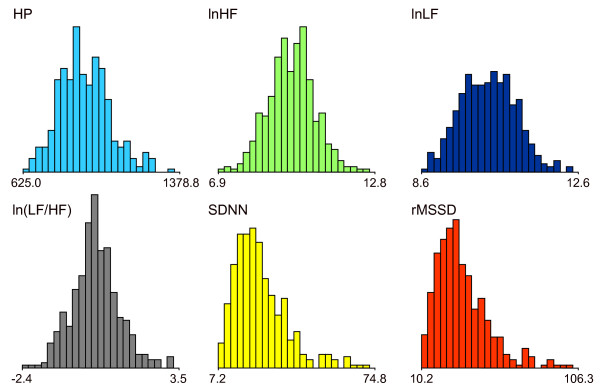
**Histograms of the interindividual variation of heart period, log-transformed heart rate variability indices and time-domain heart rate variability indices**. HRV indices included lnHF, lnLF and ln(LF/HF). Right-skewed distributions were observed for lnHF and the time-domain indices (SDNN and rMSSD).

The histograms of interindividual variations in HP and log-transformed HRV indices are shown in Figure 1. HP and log-transformed HRV indices exhibited symmetrical distributions; however, positive kurtosis (a slightly peaked curve) was observed for the distribution of ln(LF/HF) compared with the normal distribution. Regarding time-domain indices (SDNN and rMSSD), their skewness and kurtoses were high, and their distributions were considerably different from the normal distribution.

The mean, median, SD, CV, skewness, kurtosis and 5th and 95th percentiles of HRV indices are presented in Table 1. Interindividual variations (CVs) in the log-transformed HRV indices (lnHF and lnLF) were 7% to 9%; however, the time-domain indices (SDNN and rMSSD) exhibited high variabilities of 43% to 44%.

**Table 1:** Distribution characteristics of heart rate variability indices

	Heart period (ms)	lnHF (ln-ms^2^)	lnLF (ln-ms^2^)	ln(LF/HF) (ln-ratio)	SDNN (ms)	rMSSD (ms)
n	417	417	417	417	417	417
mean	945.85	9.84	10.42	0.58	27.17	37.49
median	938.86	9.89	10.42	0.56	25.09	34.76
Standard deviation^a^	126.98	0.92	0.73	0.92	11.78	16.54
Coefficient of variation (%)	13.42	9.35	7.01	-**^b^**	43.36	44.12
Skewness	0.38	0.01	0.11	0.19	1.22	1.24
Kurtosis	0.26	0.32	-0.21	0.71	1.91	2.04
Fifth percentile	748.53	8.39	9.24	-0.90	12.4	16.98
95^th ^percentile	1171.68	11.37	11.58	2.12	50.99	69.95

HF: high frequency component; LF: low frequency component; rMSSD: root mean square of successive differences; SDNN: standard deviation of normal-to-normal intervals. Skewness is a measure of symmetry of distribution. Negative or positive skewness is indicated when the left or right tail of the histogram is longer, respectively. The skewness of a normally distributed data set is zero. Kurtosis is a measure of whether the distribution curve is peaked (positive) or flat (negative) relative to the normal distribution. The kurtosis of a normally distributed data set is zero. ^a^Standard deviation of interindividual variation; ^b^the coefficient of variation of ln(LF/HF) was not calculated because it was occasionally negative.

**Figure 2:**
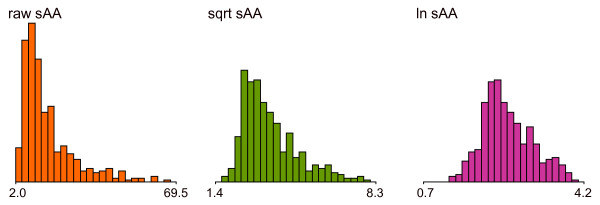
**Histograms of the interindividual variation of salivary alpha-amylase**. From left to right, the distributions of raw, square root-, and natural logarithmic-transformed sAA values are presented. The histogram of raw amylase exhibited a markedly skewed distribution. Numerical transformations (square root and natural logarithm) improved skewness, although square root transformation appeared insufficient.

Histograms of interindividual variations in raw and numerically transformed sAA values are shown in Figure 2. Extremely skewed distributions were observed for raw sAA values; square root and logarithmic transformations improved the skewness. The distribution characteristics of sAA are summarized in Table 2. Raw sAA values exhibited a large interindividual variation of approximately 67%, and square root and natural logarithmic transformations diminished this variation to approximately 20% to 30%. Numerical transformations also improved skewness and kurtosis; however, logarithmic transformation produced a better result (approximately equivalent to normal distribution), and square root transformation appeared insufficient.

**Table 2:** Distribution characteristics of raw and numerically transformed salivary alpha-amylase values

	Raw sAA	Transformed sAA	
		
	(U/mL)	Square root (sqrt [U/mL])	Natural log (ln[U/mL])
n	430	430	430
Mean	17.48	3.96	2.65
Median	13.00	3.60	2.55
Standard deviation	11.70	1.21	0.57
Coefficient of variation (%)	66.94	30.56	21.51
Skewness	1.79	1.11	0.31
Kurtosis	3.27	1.01	0.16
Fifth percentile	6.50	2.48	1.79
95^th ^percentile	43.75	6.06	3.71

The day-to-day reproducibility (ICCs) of HRV and salivary biomarker measurements are shown in Table 3. The ICC of HP was 0.67, whereas that of most HRV indices was approximately 0.5 to 0.6. Similar to the results for HRV, ICCs of raw and transformed sAA values were approximately 0.6. In contrast with skewness and kurtosis, the reproducibility of sAA measurements was not considerably improved by numerical transformations.

**Table 3:** Day-to-day reproducibility of heart rate variability and salivary alpha-amylase

	n	Intraclass correlation coefficients
Heart period	417	0.67
lnHF	417	0.59
lnLF	417	0.50
ln(LF/HF)	417	0.54
SD of normal-to-normal intervals	417	0.56
Root mean square of successive differences	417	0.58
Raw sAA	430	0.56
Square root transformed sAA	430	0.61
Natural log-transformed sAA	430	0.60

HF: high frequency component; LF: low frequency component; sAA: salivary alpha-amylase.

**Figure 3:**
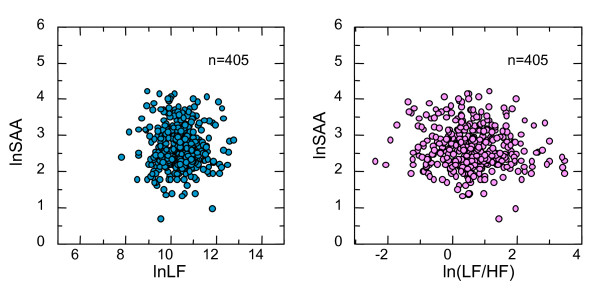
**Correlation between sympathetic indicators of heart rate variability and salivary alpha-amylase**. The left panel shows the correlation of log-transformed sAA to lnLF of HRV. The right panel shows the correlation of log-transformed sAA to ln(LF/HF) of HRV. There were no significant correlations between sAA and the sympathetic indicators of HRV.

Correlations between sympathetic indicators of HRV and log-transformed sAA measurements are presented in Figure 3. We analyzed 405 participants for whom HRV and sAA were successfully measured. Neither lnLF nor ln(LF/HF) correlated with log-transformed sAA values.

## Discussion

### Distribution characteristics of heart rate variability

Some measurements of short-term HRV in large populations have been reported; however, only a few studies focused on the interindividual distribution of these measurements. The study by Kuo *et al. *[7] is one of the few studies that reported the distribution characteristics of HRV through histograms. In addition, they demonstrated that time-domain indices exhibited similar skewed distributions; our results were consistent with these findings. In our results, positive skewness was observed in the distributions of all indices. In contrast, Kuo *et al. *[7] reported slight negative skewness in lnHF and lnLF of Taiwanese men and women aged 40 to 79 years old. The difference in this distribution might be attributed to the differences in the age and sex of the sample populations.

Many studies reported that HRV is influenced by age and sex. Both HF and LF components decrease with age, and men have relatively higher sympathetic predominance than women [7,16]. The effect of age and sex cannot be examined in our results because this study only examined healthy young men aged 20 to 29 years old.

The distributions of time-domain indices (SDNN and rMSSD) were far from normal. Compared with those in log-transformed HRV, interindividual variations in these indices were also extremely high. Therefore, when analyzing these indices, employing non-parametric tests or performing logarithmic transformation of the spectral components of HRV appears to be necessary.

### Distribution characteristics of salivary alpha-amylase

Salivary biomarkers such as amylase or cortisol can exhibit skewness in their distributions. Therefore, these values are often analyzed after logarithmic transformation. Gordis *et al. *[17] reported that the skewness for cortisol was greater than that for sAA. In according with their result, some researchers performed logarithmic transformation for cortisol and square root transformation for amylase [18,19]. In our results, the distribution curve of amylase exhibited considerable skewness. Both square root and logarithmic transformations improved the skewness of sAA; however, better results were obtained with logarithmic transformation. Square root transformation appeared insufficient.

The study by Gordis *et al. *[17] was conducted on children, whereas the present study was conducted on young men. The difference in age of the two sample populations might explain the different distributions of sAA. Further investigations are expected on the distribution of the salivary biomarker.

### Limitations for the reference values of heart rate variability and salivary alpha-amylase

The present results on HRV were calculated by MEM and thus the results are not comparable with other results calculated by fast Fourier transformation. However, the dimensionless indices such as CV, skewness and kurtosis of the distribution might be comparable with other results even if they were calculated by fast Fourier transformation.

HRV was measured during spontaneous breathing, and paced breathing was not applied in this study. Previous studies [12,20-22] reported that the LF component is decreased by paced breathing. Thus, lower LF values will be expected in the results measured during paced breathing compared with the results of this study. Paced breathing may also affect the interindividual distribution of HRV. However, a previous study reported that the effect of paced breathing on interindividual variations in the spectral components of HRV was negligible [22].

The presented values of sAA were small compared with the values reported by other studies. sAA represents apparent diurnal changes because its levels are lower in the early morning [23,24]. Raw (non-numeric transformed) sAA levels in the afternoon have been reported to be two- to three-fold higher than those in the early morning. sAA measurements of this study were performed in the early morning before breakfast. Thus, the difference is partly attributed to the time of sAA measurement. It is critical to note the time of day that sAA was measured when quantitatively examining the sAA values of different studies.

In this study, sAA was measured using a hand-held amylase monitor, and the saliva samples were analyzed immediately after saliva collection. Conversely, in most previous studies on sAA [25-28], collected saliva was frozen and stored before biochemical analysis. This difference in the measurement procedure might affect the results of sAA measurement, although it has been reported that freezing and thawing does not significantly affect sAA [18]. Further study on the effect of the device and procedure of sAA measurement is needed.

### Reproducibility of heart rate variability and salivary alpha-amylase measurements

One of the features of this study was the use of repeated measurements on different days. This permitted the analysis of the reproducibility of each HRV and salivary index. The present study appears to be the largest data set used to examine the reproducibility of short-term HRV measurements. The reproducibility (ICCs) of HRV measurements obtained in this study was slightly lower than that obtained in previous studies [12,20,21]. This may have resulted from the small age variation among the participants of this study. As mentioned previously, HRV is affected by age and thus interindividual variation in HRV decreases when age variation is narrow. A study of HRV in Japanese men aged 20 to 61 years old reported 14% to 16% interindividual variations in lnHF and lnLF [29]; conversely, the variations were 7% to 9% in this study. Roughly interpreted, ICC is the ratio of interindividual variation to total variation; therefore, ICC tends to be low when the interindividual variation is low [30].

Compared with that of HRV, the day-to-day reproducibility of sAA is not well known. Very high reproducibility (ICC = 0.92) of sAA measured using a hand-held monitor was recently reported by Aoyagi *et al. *[31]. However, sAA measurements were consecutively repeated in that study and thus did not reflect day-to-day reproducibility. Wolf *et al. *[27] reported that ICC of sAA measurements was 0.5 to 0.7 when the measurements were repeated within a span of several hours. Their result is similar to ours, although the details of the measurements were different.

### Correlation between sympathetic indicators of heart rate variability and salivary alpha-amylase

The previous studies that demonstrated a relationship between sAA and sympathetic activity (plasma norepinephrine level) examined responses to physical or mental stress [23,32], whereas the present study measured sympathetic indicators of a homogeneous population under resting condition.

If sAA is an indicator of sympathetic nervous activity, some correlation with HRV should be expected regardless of whether under resting conditions or responses to stress. In the present study, however, no correlations were observed between sAA and HRV. The interindividual variations of sAA and HRV under resting condition might not imply the variation in sympathetic tone but some other aspect of human physiology.

In addition to our results, other previous studies have also demonstrated small or insignificant correlations between sAA and sympathetic markers of HRV, although they examined the variations including responses to the various kinds of stress [11,25,27,33]. We must note that there is room for discussion on the relationship between sAA and other sympathetic indicators.

## Conclusions

Because the sample population examined in this study involved limited age and gender variations, the present results were independent of these factors and were indicative of pure interindividual variation. In other words, the interindividual variations presented in this study are the lower limit, and the variation in actual population will be larger than the values reported in this study.

## Competing interests

The authors declare that they have no competing interests.

## Authors' contributions

HK carried out the statistical analysis and interpretation of the results. BP carried out data collection and analysis. YM had overall responsibility for the study design. All authors read and approved the final manuscript.
